# Effectiveness of Carbon Localization for Invasive Breast Cancer: An Institutional Experience

**DOI:** 10.1155/2023/4082501

**Published:** 2023-07-18

**Authors:** Etienne El-Helou, Christine Eddy, Simona Picchia, Carine Van de Merckt, Magali Radermeker, Michel Moreau, Filip De Neubourg, Denis Larsimont, Isabelle Veys, C. Florin Pop

**Affiliations:** ^1^Department of Surgery, Institut Jules Bordet, Université Libre de Bruxelles, Brussels, Belgium; ^2^Department of Radiology, Institut Jules Bordet, Université Libre de Bruxelles, Brussels, Belgium; ^3^Data Centre and Statistic Department, Institut Jules Bordet, Université Libre de Bruxelles, Brussels, Belgium; ^4^Department of Pathology, Institut Jules Bordet, Université Libre de Bruxelles, Brussels, Belgium

## Abstract

**Introduction:**

The final oncological and aesthetic results of breast-conserving surgery (BCS) are influenced by the precise localization of breast cancer (BC) tumors and by the quality of the intraoperative margin assessment technique. This study aimed to assess the effectiveness of the carbon localization (CL) technique by determining the success rate of BC identification and the proportion of adequate complete resection of BC lesions.

**Methods:**

We conducted a cross-sectional retrospective study of patients treated with primary BCS for invasive BC who underwent CL of their BC lesion at the Jules Bordet Institute between January 2015 and December 2017. Descriptive statistics with categorical and continuous variables were used. The success rate of tumor identification and the rate of adequate excision were calculated using the test of percentages for independent dichotomous data.

**Results:**

This study included 542 patients with 564 nonpalpable BC lesions. The median pathological tumor size was 12 mm. Of these, 460 were invasive ductal carcinomas. Most of the tumors were of the luminal subtype. CL was performed using ultrasound guidance in 98.5% of cases. The median delay between CL and surgery was 5 days, with 46% of the patients having CL one day before surgery. The lumpectomy weighed 38 g on average, with a median diameter of the surgical sample at 6 cm and a median volume of 44 cm^3^ (6–369). One-stage complete resection was successfully performed in 93.4% of cases. In 36% of cases, an intraoperative re-excision was performed, based on intraoperative macroscopic pathological margin evaluation. The tumor was identified in 98.9% of cases in the breast surgical specimen.

**Conclusion:**

This study demonstrated high success rates for BC tumor identification (99%) and one-stage complete resection (93.4%) after BCS and CL. These results show that CL is an effective, simple, and inexpensive localization technique for successful excision of BC lesions during BCS.

## 1. Introduction

Breast cancer (BC) has become a concern for all populations, and its increasing incidence is alarming [1]. The incidence of BC increases by 0.5% each year [2] and is expected to reach more than 28 million cases in 2040 [1]. The incidence of BC varies greatly from one region to another depending on various risk factors, increases in life expectancy, and improvements in BC screening programs [3]. Improved screening strategies and early diagnosis of BC have increased the detection of hidden nonpalpable lesions [4], which are mostly often detected at an early stage and eligible for breast-conserving surgery (BCS) [5]. Since the 1990s, BCS followed by radiotherapy has become a new standard surgical procedure for these tumors, and, concomitantly, the management of BC has evolved towards the de-escalation of surgical treatment. Recent studies have demonstrated that BCS with radiotherapy not only decreases the risk of ipsilateral recurrence but is also associated with enhanced overall survival and breast-specific survival in patients undergoing BCS compared to mastectomy (Mx) [6].

To perform BCS, all cancerous tissues should be resected while preserving as much healthy tissue as possible to maintain good aesthetic outcomes and enhance patient satisfaction [5]. However, to respect these two criteria, precise localization of the tumor is necessary and a good intraoperative margin assessment technique is needed to ensure that the entire tumor is resected to avoid additional interventions [4]. Several methods for assessing intraoperative margins have been developed, based on histology, conventional radiology, and unconventional imaging techniques. In general, a negative margin for invasive BC is defined as “no ink on the tumor,” which follows the SSO-ASTRO 2014 guidelines [5].

Regarding the localization of BC tumors, wire-guided localization (WGL) has long been considered the gold standard technique for BC localization [6]. WGL involves inserting a self-retaining wire into the tumor using ultrasound (US) or stereotactic guidance [4]. This method has several drawbacks: the timing, the guidewire should ideally be positioned on the day of surgery or the day before to avoid possible wire migration and infection, the fact that the procedure requires a qualified radiologist to perform the procedure, and the potential for relatively serious postprocedural complications of wire migration and diathermy burns conducted to the skin through the wire [7]. Moreover, in addition to its complexity, reported 17% of nonpalpable cases have positive margins that require further intervention, thereby increasing the risk of recurrence [4].

Currently, multiple modalities and techniques are being developed and clinically utilized to assist in intraoperative detection of nonpalpable tumors, such as intraoperative ultrasound-guided localization (USGL) [8], intraoperative supine magnetic resonance imaging (SMRI), radioactive occult lesion localization (ROLL), radioactive seed localization (RSL), magnetic-marker localization (ML) [4], methylene blue localization (MBL) [9], cryo-assisted localization (CAL), anchor-guided localization (AGL), and indocyanine green localization (ICGL) [4].

In addition, the carbon localization (CL) procedure is another older localization technique that was first reported by Svane in 1983 [10] and has been increasingly used in recent times as an alternative procedure to WGL. CL involves the use of a sterile suspension of charcoal that is injected around the outer edges of the tumor, leaving a fine track in the parenchyma and under the skin. It is a simple, fast, and safe technique, with rare associated complications, has lower cost than that of other methods, and offers the possibility of marking at longer intervals [11].

Belgium is one of the countries with the highest incidence of BC, and for quality care, a multidisciplinary approach is always offered to patients [3]. Hence, due to the need for high-quality management of this relatively frequent pathology, CL for nonpalpable lesions has been used in our institution since the early 1990s. The aim of our study was to assess the value of CL in the preoperative workup of women diagnosed with early-stage BC undergoing BCS. We also sought to assess the effectiveness of this technique by determining the success rate of surgical excision and the proportion of adequate and complete resection of BC lesions.

## 2. Patients and Methods

### 2.1. Study Design and Patients

We conducted a cross-sectional retrospective descriptive study, covering all patients who underwent BCS for early-stage invasive BC and CL before surgical tumor resection, from January 2015 to December 2017 at the Jules Bordet Institute. Patients with a palpable tumor, those who received neoadjuvant therapy (NAT), those with only in situ disease, or those treated with mastectomy were excluded from the study. This study was conducted after review and approval by the institutional ethics committee (CE3268). The patients were identified from our prospectively maintained database review, and patient demographics, tumor characteristics, localization technique details, and treatment-type data were recorded. The characteristics of the surgical specimen (weight, maximal diameter of the specimen, volume of the excised breast specimen, complete one-stage resection, macroscopic tumor identification, and resection margins) were also collected.

### 2.2. Localization Technique: An Institutional Procedure

**Figure 1:**
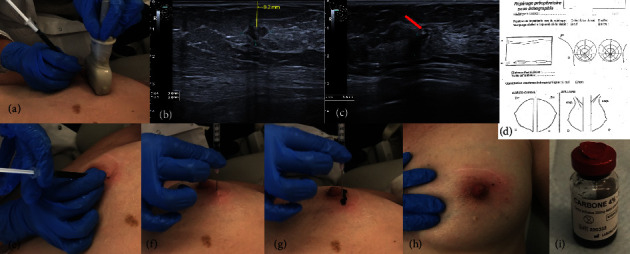
Procedural aspects of ultrasound-guided carbon localization of breast cancer lesions. The breast cancer (BC) lesion is first identified with the ultrasound (US) probe (a), and the dimensions of the tumor as well as the distance of the tumor to the skin and the position in relation to the nipple are measured (b). A syringe with an 18-gauge needle is inserted vertically (a) around the lesion usually with freehand US guidance (c), and approximately 1 mL of sterile 4% charcoal suspension is slowly injected, while the needle is withdrawn towards the skin entry point (d, f, g). Care is taken to avoid excessive carbon injection (g) just under the skin and unnecessary residual skin tattooing (h). A schematic representation (d) of the BC lesion and trace marking is made and added to the radiological report.

A few days before surgery, US was performed by one of the four institutional breast interventional radiologists to determine the location of the tumor. After identification of the lesion(s), 2-3 cc of 1% lidocaine was injected into the subcutaneous tissue overlying the lesion. A syringe with an 18-gauge needle was inserted vertically around the lesion, usually with freehand US guidance (antiparallel to the US probe), and approximately 1 mL of sterile 4% charcoal suspension (CARBONE 4%; carbo activatus 200 mg–Natrii chloridum 45 mg, Aqua p.i. ad 5 ml; Laboratoires Sterop, Brussels, Belgium) was slowly injected, while the needle was withdrawn towards the skin entry point. Care was taken to avoid excessive carbon injection just under the skin and unnecessary residual skin tattooing. After CL, a description of the tumor location, detailing the distance to the skin and the position in relation to the nipple length, was added to the radiological report, and a schematic representation of the BC lesion and trace marking was made. When the lesions were not detectable by US, CL localization was performed under stereotaxic guidance with an 18-gauge needle inserted around the lesion and/or the previously inserted marking clip. A schematic representation of the procedure is shown in Figure 1.

### 2.3. Surgical Technique and Histopathological Analysis

**Figure 2:**
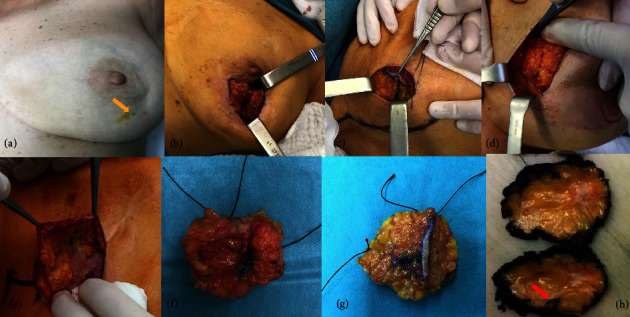
Surgical and pathological aspects of carbon localization in breast cancer tumors. During breast-conserving surgery, an incision is made over the carbon injection site (yellow arrow (a)) whenever possible. After carbon identification (b, c, e), the breast tumor lesion is excised with a macroscopic surgical margin (d). The breast surgical specimen is oriented (f, g) in a standardised way and sent to the pathology department for intraoperative gross pathological margin evaluation (h). The red arrow (h) shows the peritumoral carbon marking on the sliced surgical specimen.

During surgery, an incision was made over the carbon label injection site, whenever possible, and surgery was performed using standard BCS techniques (Figures 2(a)–2(g)). Axillary surgery was performed by sentinel lymph node biopsy (SLNB) with or without axillary lymph node dissection (ALND) according to institutional guidelines. Intraoperative margin assessment of breast surgical specimens was performed by pathological macroscopic evaluation, following current institutional practice (Figure 2(h)). A complete histopathological evaluation was then performed according to the international guidelines for BC tumor assessment.

### 2.4. Definitions

The total volume of the excised specimen was calculated with the ellipsoid formula, using the three dimensions measured by the pathologist (volume=4/3*π*(1/2length × 1/2widt h × 1/2height)). Adequate excision was defined as the complete removal of the tumor with tumor-free margins (i.e., no invasive carcinoma or ductal carcinoma in situ on inked margins). Secondary re-excision was defined as a second surgery for final positive margins (i.e., relumpectomy or mastectomy) of the same breast within 3 months. Success or failure of the localization procedure was defined by the presence or absence of a tumor in the surgical specimen after histopathological evaluation.

To assess the incidence of immediate and delayed complications after CL, physician-reported data on periprocedural (immediate) and first postoperative imaging (delayed), to detect any abnormalities correlated with the carbon injection, were collected.

### 2.5. Statistical Analysis

Statistical analysis was performed using Statistical Analysis Software (SAS). Descriptive statistics are used to summarize the patient, tumor, and clinical characteristics of the cohort. Categorical variables are reported as numbers and proportions. Continuous variables are reported as the number of subjects, mean values, standard deviation, or median and interquartile range, depending on the distribution. The effectiveness of CL in patients with nonpalpable BC was determined by evaluating the success rate of tumor identification and complete surgical removal of the tumor with adequate margins and by assessing the volume of the surgically excised breast specimen. The success rate of tumor identification and the rate of adequate excision were calculated using the test of percentages for independent dichotomous data, such as the ratio of the number of patients with a tumor (pathological) identified after BCS and the number of patients with negative margins, respectively, to the total number of cT1 patients included.

## 3. Results

### 3.1. Patients and Tumor Characteristics

This retrospective, single-institution study included 542 patients, with 564 nonpalpable invasive BC tumors, who underwent BCS after CL at the Jules Bordet Institute during the study period. The median age of the patients was 62.1 years (21.3–88.6), and the majority were postmenopausal at the time of diagnosis (84.5%). Most patients had a healthy weight, with 80.6% having BMI <25. Both sides were similarly affected (51.1% on the right side and 48.9% on the left side). Forty (7.4%) patients had multiple BC tumors, which led to excision of 564 BC tumors.  Table 1 shows the clinical and pathological characteristics of the study population. The average pathological tumor size was 12 mm (1.5–40) with only 1.8% exceeding 2 cm. Among these tumors, 460 (81.6%) were invasive ductal carcinomas (IDCs) of nonspecific type, 79 (14%) were invasive lobular carcinomas (ILCs), and the majority were positive for estrogen receptor (ER) and progesterone receptor (PR) (91.1% and 78.9%, respectively). According to the intrinsic subtype classification, most of the tumors were luminal subtype, while only 9.5% were HER2-enriched breast tumors, and 34 (6%) were triple-negative tumors. Axillary lymph nodes were involved in 15.4% of cases. These results guided adjuvant treatment following a multidisciplinary decision, consisting of intraoperative radiotherapy (IORT) and irradiation of the whole breast in 63.3% and 36.7% of cases, chemotherapy in 29.2% of cases, and endocrine therapy in 92.1% of cases.

### 3.2. Summary of the Carbon Localization Technique

Tumor localization was performed one day or more before surgery, with an average delay of 5 days between CL and surgery, using US guidance in 98.5% of cases or stereotaxic mammography in 1.5% of patients. In general, the tumors were not deep with a median tumor depth measured using US of 8 mm (Table 2).

Lumpectomy was performed for all 564 tumors mentioned above, followed by SLN biopsy in 81.4% or ALND in 17% of cases. The lumpectomy specimens weighed 38 g on average and varied from 4 g to 185 g. The average larger diameter of the sample was 6 cm and reached 15.5 cm in one case; thus, the volume of the sample varied from 6 cm^3^ to 369 cm^3^ with a median of 44 cm^3^. One-stage complete resection was successfully performed in 527 of 564 tumors, leaving behind 37 cases (6.6%) in which the final margin status was positive, leading to secondary re-excision. Additional intraoperative re-excision was performed in 36% of cases based on intraoperative macroscopic pathological margin evaluation. The tumor was identified in 562 cases (98.9% in the breast sample and 0.7% in the intraoperative re-excision sample) and was not identified in only 2 cases (0.4%) (Table 3).

### 3.3. Procedural and Postprocedural CL Complications

No allergic reactions or other important carbon-related complications were reported in our study population. Eleven patients (0.02%) developed localized hematoma. No interference was reported for pathological examination or for the first postoperative (one year) breast imaging evaluation.

## 4. Discussion

The increasing number of nonpalpable BC lesions detected by screening programs has highlighted the need for rapid and accurate localization techniques for these tumors. Our study was conducted on 564 invasive BC tumors and confirmed the efficacy of CL in correctly locating these tumors. Macroscopic identification of the tumor was successfully achieved in 98.9% of cases, including one-stage complete resection in 93.4%. In other words, in only 6.6% of cases, the final margin status was positive, slightly better than that previously reported by Svane, the godfather of this technique, who reported a rate of 7.2% of cases with positive margins [10]; however, no study has shown an improvement in this rate. This is also a clear improvement over the percentage of positive margins reported using WGL (17%) [4]. However, this low rate of final positive margins must be interpreted with caution regarding whether it is solely due to the effect of CL, considering the fact that we regularly use macroscopic pathological examination of the breast surgical specimen for the assessment of intraoperative margins.

However, this high rate of successful tumor identification in the breast surgical specimen after CL, and, consequently, the high rate of one-stage complete resection that was observed in our study, is one of the best reported rates compared to the other techniques used to localize nonpalpable BC tumors. In contrast, Davey et al. discussed in their recent review that several techniques have exhibited frustrating results with, for example, CAL, WGL, ROLL, and SMRI demonstrating positive margins in 28.2%, 20.1%, 17.2%, and 11.8% of cases, respectively [4].

Breast surgical specimen volume is another concern with regard to BCS for nonpalpable BC tumors due to its impact on postoperative breast aesthetic appearance. It is expected that BC localization techniques may help reduce resection volume and increase the final aesthetic outcome. Again, the volume of breast surgical specimens observed in our study of CL (median 44 cm^3^) is one of the smallest described, as some other techniques for BC localization have been reported to excise twice what we have reported here [12].

Carbon has long been used in skin tattooing because it is biologically inert [13], and since its application in the localization of occult breast tumors, very few publications have studied its effectiveness (Table 4 shows all PubMed-indexed English articles on CL of nonpalpable BC treated by primary surgery), and researchers have been busy finding other modernized techniques.

This was the largest study in the PubMed-indexed English literature, as reported in Table 1 [17]. Localization was performed by injecting a charcoal suspension using ultrasound in the vast majority of cases (98.5%), confirming the fact that USGL could be a BC localization technique in the future with a complementary advantage for the patient because it avoids an invasive procedure as during CL [4, 6]. However, this perioperative USGL requires special training for the breast surgeon, something which is currently missing from most basic training programs [8].

BC localization was performed the day before surgery in almost half of the cases and for up to 60 days with no tracking problems. The possibility of marking the tumor a few days before the operation facilitates preoperative preparation for both the patient and the hospital [18, 20]. Mullen et al. described successful localization performed 83 days after injection and also described the phenomenon of phagocytosis, which permanently fixes carbon particles [13].

This has also made it possible to use the CL of BC tumors even in NAT settings, with good results reported. Lannin et al. reported excellent rates of excision with free margins in patients who underwent surgery after NAT reaching 91% of cases [21]. Mathieu et al. also demonstrated that CL guided resection to the initial site of the tumor in 91% of cases without residual tumor after NAT and demonstrated carbon migration into the axillary lymph nodes in 38% of cases [22]. Although the CL approach is the localization technique currently used for BC tumors treated with NAT, these patients were excluded from the present study for more reliable results.

The pathological tumor size in our study ranged from 1.5 mm to 40 mm, which is consistent with what has been published on CL before, where the tumor size has varied between 0.7 mm and 45 mm (Table 4). It is also consistent with the tumor size reported by other existing techniques in the literature, where the mean size ranged from 8 mm to 51.3 mm [4].

No interference or disruption was reported by the pathologists at our institution during the preparation and examination of the specimens (Figure 2(h)). Cavalcanti et al. previously described the formation of an inflammatory reaction, in which they demonstrated the presence of carbon particles in the cytoplasm of macrophages in all cases, but they also confirmed that this inflammatory reaction and the presence of carbon posed no difficulty, and the pathological diagnosis was made directly [23].

No allergic reactions or carbon-related complications were reported in our study population, apart from simple complications related to the insertion of the needle itself. A few cases of carbon granuloma formation have been reported in the literature, many of which were injected and kept in place in the breast for certain reasons (e.g., benign etiology at biopsy and patient refusal) [11, 24, 25]. These have been reported to be benign formations, and no malignant transformations have been reported. In one case, granuloma formation was discovered 2 years after CL (24), confirming the inert nature of the product.

In addition to its safety and durability, one of the important reasons to use CL is its low price compared to other existing and developing BC localization modalities that require advanced instruments and technologies. Langlois and Carter demonstrated that the price of a carbon ampoule was only $2.8, while the price of wire fluctuated between $20 and $40 [14]. Similarly, Rose et al. showed that ultrasound CL after breast biopsy costs only $26.66, while hook localization costs $140 [17]. This low cost per procedural price is confirmed by our in-house ultrasound-guided BC localization technique using CL, which costs €29 at our hospital.

Different modalities and techniques are being used to help detect BC tumors [6], and each has its advantages and disadvantages, while the effectiveness of these different techniques appears to be similar. The only technique that seems to differentiate in terms of efficacy seems to be USGL, as reported in a recent meta-analysis of randomized controlled trials (RCTs) for BC tumor localization [4]. This publication mentioned that, unfortunately, the CL technique was not discussed, given the fact that there are no RCTs that compare this technique to other techniques, particularly WGL, which was a comparator for most of the RCTs of the other localization techniques currently in use.

Another relatively easy and effective concept for BC tumor localization is the ROLL technique, but its radioactive component remains an important logistical problem for a large number of hospitals [26]. To reduce the risk of diffusion of injected products, radioactive and nonradioactive “seeds” were designed: ^125^I seeds first reported in 2001, seeds with radar impulses introduced in 2014, magnetic seeds in 2016, and radiofrequency seeds in 2017. Several limitations have been described for each of these techniques [26], for which the cost remains one of the main limitations with ^125^I seed cost of almost €220 [27], whereas magnetic seeds alone can cost up to €500 [28]. Although a multitude of BC localization technique are currently available, and they demonstrate relatively similar effectiveness (successful excision rate), the evidence is limited to small cohort studies for some of these techniques, and no real comparator between the different techniques exists as most of the new techniques have been compared to WGL [4, 29].

In order to have more accurate information about the effectiveness of these techniques, the European Breast Cancer Research Association of Surgical Trialists (EUBREAST) recently launched the MELODY (methods for localization of different types of breast lesions) trial, a multinational prospective intergroup cohort study, to assess BC localization techniques and devices from several perspectives [29].

For future studies comparing these different localization techniques, careful attention must be paid to the rate of negative margins and to the standardization of intraoperative margin assessment techniques, as well as a clear definition of negative resection margins with or without intraoperative breast cavity re-excision.

Our study has limitations related primarily to its retrospective nature and the fact that we did not have the ability to compare the effectiveness of this procedure with another technique, either WGL or another technique, since we did not use another BC localization technique in our hospital. Another limitation of our study is related to the fact that, in our current procedure, we systematically used macroscopic pathological examination of the breast surgical specimen for intraoperative margin assessment, which by its nature will improve the rate of complete resection of the tumor, and not just the CL procedure itself.

## 5. Conclusion

We present this study to highlight the results of a multidisciplinary team work from our institute on the localization of nonpalpable breast tumors by CL. This was the largest study on this subject to date and demonstrated high success rates for BC tumor identification (99%) and one-stage complete resection (93.4%). These results demonstrate that CL is an effective localization technique which can guide the surgeon towards successful excision of BC lesions during BCS using a simple and inexpensive method.

## Figures and Tables

**Table 1:** Clinical and pathological characteristics of the study population.

	*N*	%
Patients	542	100
Breast tumors	564	100
Age (median) (Range)	62.1 (21.3–88.6)	
BMI (median) (Range)	24.2 (16.4–44.9)	
Affected breast		
Right	276	48.9
Left	288	51.1
Menopausal status		
Premenopausal	84	15.4
Postmenopausal	458	84.5
Histological type		
Ductal (NST)	460	81.6
Lobular	79	14.0
Other	25	4.4
Histopathological size (in mm, median) (Range)	12 (1.5–40)	
Pathological T stage		
T1	554	98.2
T2	10	1.8
Tumor grade		
1	211	37.4
2	225	39.9
3	128	22.7
Receptor-based subtype		
ER + PR + HER2−	425	75.3
ER + PR + HER2+	40	7.1
ER − PR − HER2−	34	6.0
ER − PR − HER2+	14	2.4
Pathological lymph node status		
N0	449	82.8
N1	81	14.9
N2	3	0.5
Nx	9	1.6

BMI, body mass index; NST, no specific type; ER, estrogen receptor; PR, progesterone receptor; HER2, human epidermal growth factor receptor 2; LN, lymph node; Nx, no axillary staging.

**Table 2:** Summary of the carbon localization technique.

	*N*	%
Localization method		
Ultrasound guided	534	98.5
Mammography guided	8	1.5
Timing delay of the localization procedure (in days, median) (Range)	5 (1–60)	
Timing of localization and surgery		
Previous day	250	46.1
Two days or more	292	53.9
Tumor depth on ultrasound (in mm, median) (Range)	8 (1–80)	

**Table 3:** Surgical outcomes and BC tumor excision success rates.

	*N*	%
Type of surgery		
Lumpectomy with SLNB	437	80.6
Lumpectomy with SLNB followed by ALND	64	11.8
Lumpectomy with ALND	32	5.9
Lumpectomy only	9	1.6
Complete one-stage resection		
Yes	527	93.4
No	37	6.6
Macroscopic tumor identification		
In breast surgical specimen	558	98.9
In intraoperative re-excision surgical specimen	4	0.7
Not identified	2	0.4
Weight of the breast specimen (in g, median) (Range)	38 (4–703)	
Maximal diameter of the breast specimen (in cm, median) (Range)	6 (2–15.5)	
Volume of the breast specimen (in cm^3^, median) (Range)	44 (6–368.6)	

SLNB, sentinel lymph node biopsy; ALND, axillary lymph node dissection.

**Table 4:** Summary of PubMed-indexed English publications on CL.

Year	Authors	BC lesions (N)	Imaging modality used for CL	Successful BC tumor identification (%)	Time of localization (days)	One-stage complete resection (%)	Tumor size (in mm)	Breast surgical specimen volume (median, cm^3^)
1983	Svane [10]	56	Stereotaxic	96.4	0–57	92.8	5–21	—
1991	Langlois and Carter [14]	56	Stereotaxic	—	0–42	—	1–45	—
1995	Canavese et al. [15]	158	Ultrasound (25.3%) Stereotaxic (74.7)	—	—	—	3–38	—
2001	Mullen et al. [13]	118	Stereotaxic	98.3	0–83	62.7	—	216.5
2002	Moss et al. [16]	138	Ultrasound (3.6%) Stereotaxic (96.4%)	100^*∗*^	0	76.1	—	125.6
2003	Rose et al. [17]	219	Ultrasound (48.4%) Stereotaxic (51.6%)	99	—	75.3	—	—
2007	Ko et al. [18]	109	Ultrasound	99.1	0–57	—	4–32	—
2015	Jiang et al. [19]	16	Ultrasound	100	0–3	100	—	—
2021	Zhou et al. [20]	109	Ultrasound	—	0–5	100^*∗∗*^	—	2.51
2022	This study	564	Ultrasound (98.5%) Stereotaxic (1.5%)	98.9	0–60	93.4	1.5–40	44

^*∗*^, combined WGL and CL; ^*∗∗*^, all of the breast lesions were benign.

## Data Availability

The data that support the findings of this study are available on request from the corresponding author.

## Abbreviations

AGL: Anchor-guided localization
ALND: Axillary lymph node dissection
BC: Breast cancer
BCS: Breast-conserving surgery
BMI: Body mass index
CAL: Cryo-assisted localization
CL: Carbon localization
EUBREAST: European Breast Cancer Research Association of Surgical Trialists
ER: Estrogen receptor
HER2: Human epidermal growth factor 2 receptor
ICGL: Indocyanine green localization
IDC: Invasive ductal carcinoma
ILC: Invasive lobular carcinoma
IORT: Intraoperative radiation therapy
MBL: Methylene blue localization
MELODY: Methods for localization of different types of breast lesions
ML: Magnetic-marker localization
Mx: Mastectomy
NAT: Neoadjuvant therapy
PR: Progesterone receptor
RCT: Randomized controlled trial
ROLL: Radioactive occult lesion localization
RSL: Radioactive seed localization
SLN: Sentinel lymph node
SMRI: Supine magnetic resonance imaging
SAS: Statistical Analysis Software
SSO-ASTRO: The Society of Surgical Oncology and American Society for Radiation Oncology
US: Ultrasound
UGATL: Ultrasound-guided absorbable retaining thread needle localization
USGL: Intraoperative ultrasound-guided localization
WBI: Whole breast irradiation
WGL: Wire-guided localization
$: United States dollar
€: Euro.
